# The association of vegetable consumption and physical activity with chronic obstructive pulmonary disease among community-dwelling adults aged 40 years or above in China

**DOI:** 10.3389/fnut.2025.1682112

**Published:** 2025-10-09

**Authors:** Jian Kang, Xiaojing Deng, Wenjun Wang, Hui Cheng, Yalin Hong, Huiqing Xu, Yunting Xu, Guofeng Ao, Jian Xu, Yeping Bian, Qing Ye

**Affiliations:** ^1^The First Affiliated Hospital with Nanjing Medical University, Nanjing, China; ^2^Geriatric Hospital of Nanjing Medical University, Nanjing, China; ^3^Nanjing Medical University Affiliated Nanjing Municipal Center for Disease Control and Prevention, Nanjing, China

**Keywords:** chronic obstructive pulmonary disease (COPD), vegetable consumption, physical activity, adult, Chinese

## Abstract

**Aims:**

This study aimed to examine the associations of vegetable consumption and physical activity (PA) with chronic obstructive pulmonary disease (COPD) among community-dwelling adults in regional China.

**Methods:**

Eligible participants were community-dwelling adults aged 40 years or above and were randomly selected from Nanjing municipality of China in 2023. Spirometry-based newly-identified COPD was treated as the outcome event, which was defined as post-bronchodilator FEV_1_/FVC < 0.70 and without other lung function impaired diseases, and not previously diagnosed as COPD. Independent variables were vegetable intake and PA. Logistic regression models were employed to compute odds ratio (OR) and 95% confidence interval (CI) for investigating associations of vegetable consumption and PA with COPD.

**Results:**

Among the 5,567 participants analyzed, the prevalence of spirometry-based newly-identified COPD was 14.8% (95% CI = 13.9%, 15.8%). After adjustment for socio-demographic characteristics and other potential confounding factors, participants who met vegetable consumption criteria were less likely to experience COPD compared to those who did not meet vegetable intake recommendation (OR = 0.84; 95%CI = 0.71, 0.98), while participants with sufficient PA were also less likely to experience COPD compared to physically inactive subjects (OR = 0.79; 95%CI = 0.67, 0.94). Additionally, participant who met vegetable consumption recommendation and engaged in sufficient PA were also at much lower odds to experience COPD compared to those who did not meet vegetable intake recommendation and were physically inactive (OR = 0.67; 95%CI = 0.53, 0.85).

**Conclusions:**

Vegetable consumption and PA were individually and jointly associated with CODP among community-dwelling adults in regional China. This study highlighted that, from public health perspective, intervention of vegetable consumption and PA may be of help for reducing the odds of experiencing COPD.

## Introduction

Chronic obstructive pulmonary disease (COPD) has become a significant public health concern, presently ranking the sixth leading cause of disability-adjusted life-years (DALYs) worldwide in 2021 (1). A number of classical risk factors have been documented for COPD, including: (1) tobacco smoke exposure; (2) occupational exposure to dusts, fumes or chemicals; (3) indoor air pollution; (4) early life events that prevent maximum lung growth; (5) asthma in childhood; and (6) a rare genetic condition (alpha-1 antitrypsin deficiency) (2). In addition to these established risk factors of COPD and reduced lung function, some particular lifestyle (e.g., vegetable consumption) and behaviors (e.g., physical activity [PA]) recently arose attention of researchers (3–11). Obviously, vegetable consumption and PA interventions are potential options for population-level prevention of COPD if they are examined to be influencing factors of COPD. Therefore, it is necessary to investigate the associations of vegetable consumption and PA with COPD among community-dwelling residents.

The existing literature from Western societies reported that fresh vegetable consumption was negatively associated with COPD or reduced lung function (3–6), while one from China documented that intake of vegetable was also in negative relation to self-reported COPD (7). In this Chinese study, COPD cases were self-reported but not spirometry-defined, implying that CODP cases might be under-estimated and patients' vegetable consumption and PA patterns might be intentionally modified (7). Meanwhile, PA was also negatively associated with COPD based on evidence from Western countries (8–10). Moreover, to date, the potential joint association of vegetable consumption and PA was not investigated.

In China, the nation-level prevalence of spirometry-defined COPD increased from 8.2% in 2004 to13.6% in 2015 among adults aged 40 years and older (12, 13). This highlights an urgent need to initiate effective and practicable population-level intervention programs against COPD in China. Considering that there was no literature available on the relationship between vegetable consumption, PA and spirometry-defined COPD in China, we developed this study to investigate the associations of vegetable consumption and PA with COPD among community-dwelling adults in regional China.

## Methods

### Study design and participants

This population-based cross-sectional study was conducted in Nanjing municipality of China in 2023. Nanjing municipality, the capital of Jiangsu province in China, had 9.3 million registered residents in 12 districts in 2020 (14). The inclusion criteria for eligible participants were as follows: (1) registered residents in Nanjing, (2) 40 years old or above, (3) without literal or cognitive/mental problems, (4) not experiencing any active infectious diseases, and (5) being able to do spirometry test.

Sample size calculation was based on the following factors: (1) previously-documented spirometry-based COPD prevalence (11.3%) among adults aged 40 years or above in Jiangsu province in 2019 (15), (2) cross-sectional study design, (3) multi-stage sampling approach, (4) statistical power (90%) expected, and (5) response rate (85%) assumed. Therefore, it was estimated that 5,077 participants were sufficient in this study.

Participants were randomly selected using a multi-stage sampling approach. First, eight districts were randomly determined from all the 12 districts of Nanjing municipality. Second, four streets/towns were chosen from each determined district. Third, one or two neighborhoods/villages were selected from each chosen street/town according to the street/town-district proportion of residents aged 40 years or above. Fourth, 60 households were recruited from each selected neighborhood/village. Fifth, all residents aged 40 years or above in the chosen households were invited to take part in questionnaire survey and spirometry test. Finally, 6,403 eligible participants were selected for this study.

Informed consent was obtained from all participants before the survey. This study was reviewed and approved by the Ethics Committee of the Nanjing Medical University Affiliated Nanjing Municipal Center for Disease Control and Prevention. Study methods were in line the recommended principles in the Declaration of Helsinki. Additionally, as only de-identified data analyzed in the present study, the secondary ethical approval was waived by the Ethics Committee of Geriatric Hospital of Nanjing Medical University.

### Data collection

Data were gathered using a questionnaire, the Chinese Adult Chronic Disease and Nutrition Surveillance Questionnaire, developed by Chinese Center for Disease Control and Prevention (CCDC) (16). The following information was collected from participants: socio-demographic attributes (age, sex, location, educational level, marital status, and income), potential influencing factors of COPD (vegetable consumption, PA, smoking status, exposure history of environmental and occupational factors), personal history of selected chronic diseases, and family history of COPD. The participants were interviewed by research team members in local community health service centers.

### Lung function test

Lung function was tested for participants by physicians from local community health service centers using portable spirometers (Flow Screen, Jaeger, Germany). Prior to the study, all the selected physicians received training to learn how to adequately operate portable spirometers. Only after passing qualification examination, these physicians would be responsible for lung function test in the study. Lung function test procedures were as follows: (1) Firstly, all participants received baseline spirometry tests; (2) Then, for participants who had no contraindication of salbutamol aerosol, they inhaled 400 μg of salbutamol aerosol, and, 15 min later, received post-bronchodilator spirometry tests. Forced expiratory volume in one second (FEV_1_) and forced vital capacity (FVC) were used to assess lung function for each participant.

### Outcome variables

The outcome event was newly-identified COPD. Participants were defined as newly-identified COPD cases (13), if they: (1) had a post-bronchodilator FEV_1_/FVC < 70%, and without other lung-function-impaired conditions (asthma, lung cancer, pulmonary heart disease, tuberculosis, lung surgery or musculoskeletal diseases); and (2) have not already been diagnosed with COPD by a respiratory physician. In analysis, participants were classified as “with newly-identified COPD” or “without COPD”.

### Independent variables

Vegetable consumption and PA were the two independent variables. Chinese Nutrition Society (CNS) released different recommendations of vegetable consumption for general and older Chinese adults: consumption frequencies and amount for general adults, whereas consumption frequency for older adults (17, 18). Additionally, this study was developed primarily for investigating COPD among adults aged 40 years and older. Only selected items from a validated food frequency questionnaire (FFQ) were employed to assess food consumption (19). Information on vegetable consumption frequency was gathered using the following item in this FFQ: How often did you consume fresh vegetable under a typical situation in last year? The answer option was: Please specify the consumption frequencies (19). Intake frequencies were available for both general and older adults in the study. Therefore, to ensure the consistency in assessing vegetable consumption for all participants, frequency was used to measure intake level of vegetable. The frequency of vegetable consumption was converted into weekly times for each participant. CNS recommended that a typical Chinese adult shall consume at least 14 times of fresh vegetable per week (17, 18). Using this cutoff, participants were classified as “recommended level reached” or “recommendation not reached” in the analysis.

The past seven-day PA was measured using the Chinese version of International Physical Activity Questionnaire (IPAQ-CHN) for each participant (20). Moderate and vigorous PA levels in last seven days were assessed separately, and then the weekly time of moderate plus doubled vigorous PA (MVPA) was computed for classifying participants into: “sufficient (MVPA ≥ 150 min/7 days) ” or “insufficient (MVPA < 150 min/7 days)” PA (21).

To analyze the joint association of vegetable consumption and PA with COPD, participants were also categorized into four sub-groups: “vegetable consumption recommendation not reached + insufficient PA” (the reference), “vegetable consumption recommendation reached + insufficient PA”, “vegetable consumption recommendation not reached + sufficient PA”, or “vegetable consumption recommendation reached + sufficient PA”.

### Covariates

Socio-demographic attributes and other potential risk factors of COPD were considered in the analysis. Socio-demographic characteristics referred to age (40–59, 60–79 or 80+ years), sex (man or woman), location (urban or sub-urban), educational level ( ≤ 9, 10–12 or ≥13 years of schooling), marital status (married/having a partner or no spouse/partner), and household-based income per capita (tertiled as lower [ < ¥15,600], middle [¥15,600–33,332] or upper [≥¥33,333]). Potential risk factors of COPD analyzed in this study included active and passive smoking status, meat consumption, exposure history of cooking environmental and occupational factors, personal history of diabetes and hypertension, and family history of COPD. They were measured through self-report using definitions and classifications adopted from the Scheme of Chinese chronic non-communicable disease and risk factor surveillance released by CCDC (16). Participants were classified as current, former or never smokers according to active smoking history, and alcohol drinkers or non-drinkers (16). Passive smoking referred to an exposure to tobacco smoke for ≥15 min/day at home or in the workplace (16). The intake frequencies of red meat and white meat were self-reported by participants using selected items from the FFQ (19). CNS did not recommend separate consumption level of red and white meat, but just simply recommended to consume 7–14 times/week of meat (combination of red and white meat) for Chinese adults (17, 18). Therefore, based on intake frequency of combination of red and white meat, participants were classified into three categories: less than recommendation, recommendation reached, or over recommendation. Biomass fuels referred to coal or wood/straw. History of exposure to biomass fuels was measured using three question items: (1) in the past year, were you usually responsible for cooking at home? (2) what primary fuel used for cooking in your family (four options: natural gas, electricity, coal, or wood/straw)? and (3) when preparing food in kitchen, did you use hood/fan to exhale kitchen air? Based on these three items, if participants prepared cooking typically with biomass fuels and not using kitchen hood/fan, they were classified as “positive exposure to biomass fuels during cooking”. Otherwise, they were categorized as “no exposure to biomass fuels” (16). Subjects were also grouped into: “positive occupational exposure to dust/harmful gases during work or agricultural production” or “no such an exposure” (16). According to self-reported history of diabetes and hypertension, participants were classified as “having diabetes/hypertension” or “not having diabetes/hypertension” (16). Positive family history of COPD was defined as that at least one parent had already been diagnosed with COPD (16).

With light clothing and no shoes, participants were measured two times to record their body weight and height, separately. The mean readings of height and weight were used to calculate body mass index (BMI) as weight (kg) divided by height squared (m^2^). Then, based on BMI cutoffs recommended for Chinese adults (22), participants were classified into: underweight (BMI < 18.5 kg/m^2^), normal (18.5 ≤ BMI < 24 kg/m^2^) or overweight/obese (BMI≥24 kg/m^2^).

### Statistical analysis

First, data were presented as categorical variables (%). Chi-square test was conducted to examine differences in COPD prevalence between participants with different characteristics. Then, three mixed-effect logistic regression models were employed to compute odds ratios (ORs) and 95% confidence intervals (95% CIs) for investigating the associations of vegetable consumption and PA with COPD. Model 1 was an unadjusted analysis with vegetable consumption or PA as the single predictor and adjustment for neighborhood-level clustering effect. Model 2 was a multivariable analysis with adjustment for socio-demographic characteristics (age, sex, area, educational level, marital status, and annual income) in addition to those in Model 1. Model 3 was also a multivariable analysis with further adjustment for body weight status, PA (where applicable), vegetable consumption (where applicable), meat intake, active smoking, passive smoking, drinking, history of exposure to polluted fuels, history of occupational exposure, diabetes, hypertension, and family history of COPD in addition to those in Model 2. Significance level was set as *p* < 0.05 (two-sided). Data were managed and analyzed using SPSS 23.0 (SPSS Inc., Chicago, IL, USA).

## Results

### Characteristics of participants

Table 1 demonstrates the selected characteristics of participants by vegetable consumption and PA in this study. Among the 6,403 participants selected, 5,840 were successfully recruited (91.2%). Of the 563 residents who did not respond to the study, 241 refused to take part in and 322 were not accessible at the appointed survey date. Additionally, 235 participants were not able to complete spirometry test due to contraindications of salbutamol. Among the 5,605 participants completed lung function test, 38 were already-diagnosed COPD patients and therefore excluded from analysis. Finally, 5,567 participants were included in this study, and their mean age was 59.3 years (standard deviation = 9.6). Moreover, 47.0% were men, and 38.8% were urban residents. There were 60.7% and 76.8% of participants meeting the criteria of vegetable consumption and sufficient PA, separately. There were significant differences in proportions of participants who met recommendation of vegetable consumption and PA between age, sex, and area, respectively.

**Table 1:** Selected socio-demographic characteristics of participants by vegetable consumption and physical activity in this study.

**Characteristics**	**Overall**	**% (** ***n*** **) of participants meeting recommendation**			
		**Vegetable consumption** ^*^	***p*** **-value**	**Physical activity** ^#^	***p*** **-value**
Overall	5,567	60.7 (3,377)		76.8 (4,274)	
**Age group (years)**					
40–49	15.7 (876)	16.9 (572)		15.0 (639)	
50–59	36.6 (2,037)	38.0 (1,285)	< 0.001	37.3 (1,594)	< 0.001
60–69	32.7 (1,820)	31.7 (1,069)		33.9 (1,451)	
70–79	13.5 (750)	12.1 (408)		12.6 (539)	
≥80	1.5 (84)	1.3 (43)		1.2 (51)	
**Sex**					
Man	47.0 (2,616)	47.0 (1,586)	0.961	45.0 (1,924)	< 0.001
Woman	53.0 (2,951)	530 (1,791)		55.0 (2,350)	
**Area**					
Urban	38.8 (2,158)	32.0 (1,081)	< 0.001	40.2 (1,720)	< 0.001
Suburban	61.2 (3,409)	68.0 (2,296)		59.8 (2,554)	
**Education (schooling years)**					
≤ 9	39.7 (2,209)	42.6 (1,439)		39.4 (1,685)	
10–12	33.5 (1,866)	32.9 (1,112)	< 0.001	33.3 (1,424)	0.375
≥13	26.8 (1,492)	24.5 (826)		27.3 (1,165)	
**Marital status**					
Single	7.2 (400)	7.5 (252)	0.320	7.4 (317)	0.223
Married	92.8 (5,167)	92.5 (3,125)		92.6 (3,957)	
**Average income per capita (tertiled)**					
Lower	32.2 (1,793)	33.0 (1,113)		32.4 (1,386)	
Middle	33.7 (1,875)	32.7 (1,105)	0.138	33.7 (1,437)	0.803
Upper	34.1 (1,899)	34.3 (1,159)		33.9 (1,451)	

^*^Vegetable consumption was categorized into: “recommendation reached” or “recommendation not reached” based on corresponding consumption recommendations for Chinese adults by Chinese Nutrition Society 2016.

^#^Physical activity (PA) was classified as “sufficient PA (≥150 min/week)” or “insufficient PA (< 150 min/week)” based on the total time spent in moderate PA plus twice the time spent in vigorous PA during the past 7 days assessed using the International Physical Activity Questionnaire.

### Prevalence of newly-identified COPD

Table 2 presents the prevalence of spirometry-based newly-identified COPD. Totally, 825 participants were identified as newly-identified COPD patients, with an overall prevalence of 14.8% (95% CI = 13.9%, 15.8%) among 5,567 participants. The prevalence of COPD was 8.3%, 12.6%, 16.9%, 21.7% and 31.0% among adults aged 40–49, 50–59, 60–69, 70–79 and 80+ years, respectively. Moreover, the prevalence of COPD decreased with either educational attainment or average income increasing. Additionally, the prevalence of COPD was significantly higher among single adults or those with exposure to biomass fuels compared to their counterparts, accordingly.

**Table 2:** Prevalence of spirometry-based newly-identified COPD by selected factors among participants in this study.

**Characteristics**	**% (** ***n*** **) of participants**			**Chi-square**	***p*-value**
	**Overall**	**Non-COPD**	**COPD** ^*^		
Overall	5,567	85.2 (4,742)	14.8 (825)		
**Age (years)**					
40–49	15.7 (876)	16.9 (803)	8.8 (73)		
50–59	36.6 (2,037)	37.6 (1,781)	31.0 (256)		
60–69	32.7 (1,820)	31.9 (1,513)	37.2 (307)	89.15	< 0.001
70–79	13.5 (750)	12.4 (587)	19.8 (163)		
≥80	1.5 (84)	1.2 (58)	3.2 (26)		
**Sex**					
Man	47.0 (2,616)	47.2 (2,240)	45.6 (376)	0.78	0.377
Woman	53.0 (2,951)	52.8 (2,502)	54.4 (449)		
**Area**					
Urban	38.8 (2,158)	38.5 (1,828)	40.0 (330)	0.62	0.430
Suburban	61.2 (3,409)	61.5 (2,914)	60.0 (495)		
**Education (schooling years)**					
≤ 9	39.7 (2,209)	38.2 (1,813)	48.0 (396)		
10–12	33.5 (1,866)	33.9 (1,607)	31.4 (259)	32.07	< 0.001
≥13	26.8 (1,492)	27.9 (1,322)	20.6 (170)		
**Marital status**					
Single	7.2 (400)	6.5 (310)	10.9 (90)	20.14	< 0.001
Married	92.8 (5,167)	93.5 (4,432)	89.1 (735)		
**Average income per capita (tertiled)**					
Lower	32.2 (1,793)	31.0 (1,472)	38.9 (321)		
Middle	33.7 (1,875)	33.6 (1,593)	34.2 (282)	28.31	< 0.001
Upper	34.1 (1,899)	35.4 (1,677)	26.9 (222)		
**Family history of COPD** ^$^					
No	97.3 (5,415)	97.3 (4,613)	97.2 (802)	0.01	0.913
Yes	2.7 (152)	2.7 (129)	2.8 (23)		
**Smoking status** ^†^					
Never-smokers	72.1 (4,016)	72.0 (3,412)	73.2 (604)		
Current smokers	22.6 (1,259)	22.9 (1,084)	21.2 (175)	1.20	0.549
Former smokers	5.2 (292)	5.1 (246)	5.6 (46)		
**Passive smoking** ^‡^					
No	64.7 (3,628)	65.5 (3,057)	65.5 (540)	0.22	0.636
Yes	35.3 (1,977)	35.5 (1,685)	35.5 (285)		
**BMI category (kg/m** ^2^ **)** ^¶^					
< 18.5	1.3 (70)	1.2 (56)	1.7 (14)		
18.5–23.9	36.2 (2,016)	35.6 (1,688)	39.8 (328)	7.82	0.050
24–27.9	44.3 (2,466)	44.6 (2,117)	42.3 (349)		
≥28	18.2 (1,015)	18.6 (881)	16.2 (134)		
**History of exposure to polluted fuels**					
No	96.7 (5,386)	97.0 (4,599)	95.4 (787)	5.65	0.017
Yes	3.3 (181)	3.0 (143)	4.6 (38)		

^*^Newly-identified COPD patients referred to individuals who were not previously diagnosed with COPD, but had a spirometer-measured FEV1/FVC < 70% and no other lung-function-impaired diseases (such as asthma, tuberculosis, lung cancer, pulmonary heart disease, lung surgery or musculoskeletal diseases).

^$^Positive family history of COPD referred to that at least one parent had been diagnosed as COPD patient.

^†^Smoking status was defined as smokers (current- and ex-smokers) and non-smokers (who never smoked cigarettes).

^‡^Passive smoking was defined as an exposure to second-hand tobacco smoke for at least 15 min/day at home or in the workplace.

^¶^BMI referred to body mass index, which was used to define participant's body weight status based on cutoffs recommended for Chinese adults.

### The association of vegetable consumption and PA with newly-identified COPD

Table 3 shows the associations of vegetable consumption and PA with newly-identified COPD among participants. After adjustment for socio-demographic characteristics and other potential influencing factors, participants meeting criteria of vegetable consumption were at significantly lower odds to have COPD (OR = 0.84; 95%CI = 0.71, 0.98) relative to their counterparts who did not meet vegetable intake recommendation, while participants with sufficient PA were also less likely to experience COPD (OR = 0.79; 95%CI = 0.67, 0.94) compared to those with insufficient PA.

**Table 3:** The associations of vegetable consumption and physical activity with spirometry-based newly-identified COPD among participants in this study.

**Exposure variable**		**Participant number, *N***	**Prevalence of newly-identified COPD✩, % (*n*)**	**OR (95%CI) for experiencing COPD** ^**$**^		
**Vegetable consumption** ^*^	**Physical activity** ^#^			**Model 1** ^†^	**Model 2** ^‡^	**Model 3** ^¶^
Recommendation not reached		2,190	16.6 (363)	1	1	1
Recommendation reached		3,377	13.7 (462)	0.80 (0.69, 0.93)	0.82 (0.70, 0.96)	0.84 (0.71, 0.98)
	Insufficient	1,293	17.4 (225)	1	1	1
	Sufficient	4,274	14.0 (600)	0.78 (0.66, 0.92)	0.78 (0.66, 0.93)	0.79 (0.67, 0.94)
Recommendation not reached	Insufficient	623	18.8 (117)	1	1	1
	Sufficient	1,567	15.7 (246)	0.81 (0.63, 1.03)	0.83 (0.65, 1.06)	0.81 (0.63, 1.04)
Recommendation reached	Insufficient	670	16.1 (108)	0.83 (0.62, 1.11)	0.87 (0.65, 1.17)	0.87 (0.65, 1.17)
	Sufficient	2,707	13.1 (354)	0.65 (0.52, 0.82)	0.68 (0.54, 0.86)	0.67 (0.53, 0.85)

^*^Vegetable consumption was categorized into: “recommendation reached” or “recommendation not reached” based on corresponding consumption recommendations for Chinese adults by Chinese Nutrition Society 2016.

^#^Physical activity (PA) was classified as “sufficient PA (≥150 min/week)” or “insufficient PA (< 150 min/week)” based on the total time spent in moderate PA plus twice the time spent in vigorous PA during the past 7 days assessed using the International Physical Activity Questionnaire.

✩ Newly-identified COPD patients referred to individuals who were not previously diagnosed with COPD, but had a spirometer-measured FEV1/FVC < 70% and no other lung-function-impaired diseases (such as asthma, tuberculosis, lung cancer, pulmonary heart disease, lung surgery or musculoskeletal diseases).

^$^OR, odds ratio; CI, confidence interval.

^†^Model 1 was an unadjusted mixed-effects logistic regression analysis with vegetable consumption as the single predictor and adjustment for neighborhood-level clustering effects.

^‡^Model 2 was a multivariable mixed-effect logistics regression analysis with adjustment for socio-demographic characteristics (age, sex, area, educational level, marital status, annual income), in addition to those in Model 1.

^¶^Model 3 was a multivariable mixed-effect logistics regression analysis with further adjustment for body weight status, physical activity (where applicable), vegetable consumption (where applicable), meat consumption, family history of COPD, active smoking, passive smoking, drinking, history of exposure to polluted fuels, history of occupational exposure, diabetes, and hypertension in addition to those in Model 2.

With regard to joint association of vegetable consumption and PA with COPD, after control for socio-demographic attributes and other potential confounding factors, the odds for experiencing COPD were not significantly different between participants with “vegetable consumption recommendation not reached and sufficient PA” (OR = 0.81; 95%CI = 0.63, 1.04) or “recommendation of vegetable consumption reached and insufficient PA” (OR = 0.87; 95%CI = 0.65, 1.17) and the reference group (participants with “less vegetable consumption and insufficient PA”). However, notably, compared to those with “vegetable intake recommendation not reached and insufficient PA”, participants with “recommendation of vegetable consumption reached and sufficient PA” were at a significantly lower odds to experience COPD (OR = 0.67; 95%CI = 0.53, 0.85).

## Discussion

The primary aim of this population-based study was to investigate the association of vegetable consumption and PA with newly-identified COPD among community-dwelling adults in regional China. The prevalence of spirometry-based newly-identified COPD was 14.8%. Vegetable consumption and PA were each in significantly negative relation to COPD. Moreover, these two factors were also jointly associated with COPD among adults aged 40 years or above in China.

The negative relationship between vegetable consumption and COPD observed in this study is in line with previous reports (3–7). Among the very few original studies documenting relationship between vegetable intake and COPD/lung function (5–7), two were from Western countries (5, 6), and one was from China (7). Consumption frequencies were used as the main explanatory measure in all these existing studies (5–7). In our study, consumption frequency was also used to measure vegetable intake level for participants, which could ensure comparability between findings in the previous studies and ours. However, the designs were different for the previous studies, including two cohort studies in Western countries (one in Sweden, the other in the UK) (5, 6), one cross-sectional survey from China (7), and two meta-analysis/systematic reviews (3, 4), whereas our study was a cross-sectional investigation. Moreover, in the two cohort studies, daily or weekly serving frequencies of vegetable intake were categorized into quintiles, while COPD and lung function were used as the outcome variables in the Sweden and UK studies, respectively (5, 6). The Sweden study included 44,335 men (aged 45–79 years) and found that participants in the highest quintile of total vegetable consumption were at a significantly lower risk (OR = 0.82, 95%CI = 0.70, 0.97) to develop COPD compared to those in the lowest quintile (5). The UK study had 2,942 adults (mean age: 65.7 and 66.6 for men and women) and found that vegetable and fruit intake was positively associated with lung function (6). Additionally, fresh vegetable was analyzed in studies from Western Countries (5, 6), while cooked-vegetable was analyzed in the previous Chinese study (7) and ours. Notably, the outcome events were different in the previous Chinese study and ours: self-reported COPD patients were treated as outcome events in the previous Chinese study (7), whereas only spirometry-based newly-identified COPD cases were analyzed in ours. Therefore, evidence from studies with different design can each other consistently reinforce the inverse relationship between vegetable consumption and COPD.

The negative association of PA with COPD found in our study aligns with that reported in existing literature (8, 9). To date, only two studies on PA-COPD relationship were available and all were cohort studies: one using the UK Biobank data and the other using prospective cohort data from Denmark (8, 9). In the study with UK Biobank data, PA was measured using IPAQ (8), whereas PA was indicated with physical fitness in Denmark cohort study (9). However, these two studies consistently highlighted a negative association of PA with COPD among community-based adults (8, 9). In our study, the IPAQ (Chinese version) was employed to assess PA for participants. Therefore, the negative association between PA and COPD may hold true irrespective of measures of PA.

Although very few studies were available, almost all of them were designed as cohort studies, which could imply causal associations of vegetable intake and PA with COPD among adults in Western countries (3–6, 8, 9). The previous Chinese study, a cross-sectional survey with self-reported COPD as outcome event, could not provide any causality between vegetable intake and COPD in Chinese adults (7). However, our study, a cross-sectional survey with only newly-identified COPD cases included in analysis, might yield somewhat causal associations of vegetable consumption and PA with COPD for Chinese adults, as participants might not intentionally modify the COPD-related lifestyle. This is what our study significantly added to existing literature regarding associations of vegetable intake, PA with CODP.

It is known that oxidative stress and inflammation are the major driving factors of COPD (23, 24). Vegetable consumption may have the ability to reduce levels of oxidative stress (25) and inflammation parameters (26), increase levels of antioxidant defense (25). This is plausibly due to that vitamin C (27, 28), β-carotene (29), and dietary fibers (30) contained in vegetable may play the role in reducing levels of both oxidative stress (27–30) and inflammatory biomarkers (26, 31). Moreover, vitamin C and β-carotene intake may be also positively associated with lung function (29, 32).

The negative association between PA and COPD may also be, at least partly, explained by the positive effect of PA on inflammation (33–37). It has been documented that physical inactivity was positively associated with increased levels of inflammation (measured with high-sensitivity C-reactive protein, interleukin 1 and 6, or fibrinogen) and oxidative stress (34–36). In contrast, sufficient PA may reduce both inflammation and oxidative stress (33, 36). Considering that both vegetable intake and PA share the same pathway to influence the development of COPD, it is reasonably assumed that vegetable consumption and PA may exert joint influence on COPD through reducing systemic inflammation and oxidative stress.

Although the primary purpose of this study was to investigate the association of vegetable intake and PA with newly-identified COPD, it is still of interest to take a look at the prevalence of already-diagnosed COPD. Among the initial 5,605 overall participants, a total of 863 COPD individuals were identified. However, only 38 participants were those already-diagnosed cases, showing a very low proportion of already-diagnosed COPD individuals (4.4% = 38/863). This suggested that already-diagnosed COPD patients were just the tip of the iceberg. Moreover, this also implied that most COPD individuals (the un-diagnosed) did not seek clinical treatment or lifestyle/behavior modification intentionally as they did not know experiencing COPD. These highlight that substantial burden would be caused by COPD in future. Therefore, to reduce the potentially substantial COPD burden, it is critically important to initiate population-based COPD screening programs and provide accessible and affordable treatment for COPD patients.

This study had several strengths. First, this is the first study that examined the associations of vegetable consumption and PA with COPD among community-dwelling adults in China. Second, objectively-measured lung function test was used to determine COPD cases. Third, only new-identified COPD cases were treated as outcome events in analysis, ensuring that participants' vegetable consumption and PA patterns were unlikely to have been intentionally modified due to a COPD diagnosis. Therefore, to some extent, the relationship between vegetable consumption, PA and COPD observed in this study was of causal direction. Fourth, classical confounding factors associated with COPD—including socio-demographic attributes, body weight status, lifestyle and behaviors, environmental risk factors, selected chronic diseases, and family history of COPD—were controlled for in the analysis.

Some limitations were worthy of mention in this study. First, vegetable consumption and PA level were self-reported by participants, which might introduce potential recall bias, although they were measured using validated instruments, separately. Second, data on socio-demographic attributes, lifestyle and behaviors, environmental and occupational risk factors, selected chronic diseases, and family history of COPD were also self-reported by participants, implying potential bias too. Fourth, histories of early life events and childhood asthma, two established risk factors of COPD, were not considered in analysis due to lack of data. Fifth, a skewed distribution was examined for vegetable intake, which could not allow us to use it as a continuous measure investigating the possible dose-dependent association between vegetable intake and COPD. Sixth, compared to intake amount, consumption frequency of vegetable was less informative. For participants sharing the same vegetable intake frequency, they might actually consumed different amount of vegetable if the serving amount was different. However, unfortunately, due to lack of data, we could not use intake amount of vegetable or calculate energy intake in the analysis. The last, portable spirometers were used to test participants' lung function in this study. Although portable spirometers may not perform as well as those used for clinical diagnosis of COPD in respiratory medicine, it has been examined that their performance was acceptable compared to those used in clinical practice (38). Moreover, lung function was assessed with portable not clinically-conventional spirometers, resulting in that newly-identified COPD cases were not clinically-diagnosed patients. In future, it is encouraged to further investigate the associations of vegetable or other foods intake, and PA with the risk of developing COPD for population-level COPD prevention.

In conclusion, vegetable consumption and PA were individually and jointly associated with CODP among community-dwelling adults in regional China. Considering the burden caused by COPD, it shall be the priority to target population-level intervention programs for COPD prevention in addition to treatment of patients. This study highlighted that, from public health perspective, intervention of vegetable consumption and PA may be of help for reducing the odds of experiencing COPD.

## Data Availability

The raw data supporting the conclusions of this article will be made available by the authors, without undue reservation.
